# Genetic and potential non-genetic benefits increase offspring fitness of polyandrous females in non-resource based mating system

**DOI:** 10.1186/1471-2148-10-20

**Published:** 2010-01-22

**Authors:** Jukka Kekäläinen, Geir Rudolfsen, Matti Janhunen, Lars Figenschou, Nina Peuhkuri, Niina Tamper, Raine Kortet

**Affiliations:** 1 Department of Biological and Environmental Science, University of Jyväskylä, P.O. Box 35 (YAC-315.2), FI-40014 University of Jyväskylä, Jyväskylä, Finland; 2 Ecological Research Institute, University of Eastern Finland, P.O. Box 111, FI-80101 Joensuu, Finland; 3 Department of Evolution and Ecology, Institute of Biology, University of Tromsø, N- 9037 Tromsø, Norway; 4 Finnish Game and Fisheries Research Institute, Joensuu Game and Fisheries Research, Yliopistonkatu 6, FI-80100 Joensuu, Finland; 5 Finnish Game and Fisheries Research Institute, Viikinkaari 4, P.O. Box 2, FI-00791 Helsinki, Finland; 6 Department of Biology, University of Eastern Finland, P.O. Box 111, FI-80101 Joensuu, Finland; 7 Department of Biology, University of Oulu, P.O. Box 3000, FI-90014 University of Oulu, Oulu, Finland

## Abstract

**Background:**

The adaptive significance of female polyandry is currently under considerable debate. In non-resource based mating systems, indirect, i.e. genetic benefits have been proposed to be responsible for the fitness gain from polyandry. We studied the benefits of polyandry in the Arctic charr (*Salvelinus alpinus*) using an experimental design in which the material investments by the sires and maternal environmental effects were controlled.

**Results:**

Embryonic mortality showed a strong paternal genetic component, and it was lower in polyandrously fertilized offspring (sperm competition of two males) than in monandrous fertilizations. We also found that high sperm velocity was associated with low offspring mortality, but not with the size of the offspring or their yolk volume. Although no male effect was found on the size of the offspring yolk reserves, yolk volume was higher in offspring from polyandrous matings than offspring of the either of the two males when mated monandrously.

**Conclusions:**

In support of the "good sperm hypothesis, we found that sperm velocity was positively associated with offspring fitness. In addition, our results suggest that polyandrous females gain genetic advantage (higher offspring survival) from this behavior, but that some benefits of polyandry (larger yolk volume) may not be explained solely by the additive genetic effects. This suggests that sperm competition environment may intensify the selection on genetically superior sperm which in turn may produce offspring that have superior yolk reserves. However, as high sperm velocity was not associated with larger yolk volume, it is possible that also some other non-genetic effects may contribute to offspring fitness. The potential role of polyandrous mating in inbreeding avoidance is discussed.

## Background

Mating with several males (polyandry) [1-9] can incur various costs to the females [10]. Mostly for this reason, the adaptive significance of this behaviour has been under considerable debate. When females gain no direct material benefits from mating, indirect (i.e. genetic) advantages are believed to explain the evolution of polyandry [8,11-15] (see also [16]). By mating with many males, females may increase the probability of their eggs becoming fertilized by the sperm of genetically superior or compatible male [3,17]. Thus, certain genes or gene combinations should result in polyandrous females producing offspring of higher fitness than monandrous females.

"Good sperm" hypothesis predicts that sperm that are more successful in competition for fertilising the eggs are also more effective in producing viable offspring. In other words, a male's sperm competition ability should correlate with the fitness of his offspring [18-21]. Previous studies that have demonstrated genetic benefits of polyandry have often been confounded by differential maternal investments (reviewed by [8]). Accordingly, the empirical evidence supporting the "good sperm" hypothesis is scarce [22-24]. Hosken *et al*. [24] found that males that were more successful in sperm competition had offspring that developed faster than offspring of males with lower quality sperm. However, no association between offspring mortality and the competitive ability of sperm was detected. To the best of our knowledge, only one study, with the Australian marsupial (*Antechinus stuartii*) has demonstrated that males gaining high paternity under sperm competition (i.e. good sperm competitors) also sire offspring with lower mortality rate [25]. Although the genetic female benefits of polyandry have been demonstrated in numerous studies, some recent studies have also shown that the benefits of polyandry may not always be transmitted through conventional additive genetic pathways [15,26,27] (see also [28,29]). In addition, benefits of polyandry may depend on certain female characteristics (e.g. condition), which suggest that genetic benefits are not necessarily equal to all individuals [15].

In many oviparous fish with no parental care, starvation during the critical transition period from endogenous feeding to independent foraging is among the most important causes of mortality in the course of the ontogeny (e.g. [30-33]). Thus, the development of newly hatched larvae is highly dependent on yolk reserves [32], larger reserves giving more time to initiate external feeding before exhaustion [34]. In general, the amount of yolk may be critical to the survival of the offspring [35]. The size of the egg and yolk has often been considered solely as a female dependent factor and the possible male effects have often been neglected [36]. However, the male may contribute to egg size after fertilization by affecting the water uptake of the eggs [36] (see also [37,38]). In addition, males can also affect to the size of the offspring (or yolk) indirectly, through metabolic rate of the embryo [38] (see also [39]). Hence, males could indirectly contribute to progeny quality and survival, even in species that provide no parental care.

The Arctic charr (*Salvelinus alpinus*) has a lek-like breeding system, where both sexes mate multiply. During egg release one large male is usually in close contact with the female [40] and generally fertilizes the greatest proportion of the eggs [41]. However, several smaller males are usually also present in the vicinity of the female and these sneaking males fertilize a proportion of the eggs leading to intense sperm competition between males [42]. Thus, two types of polyandrous mating occur: First, females mate with different males in different spawning acts, and second, several males compete for fertilizing eggs within single act.

As the "good sperm" hypothesis predicts, sperm velocity is the most important factor predicting the paternity of the male Arctic charr, and also in other salmonids [43,44] (but see [45]). On the other hand, ovarian fluid affects the velocity and longevity of charr sperm [46], indicating that cryptic female choice may be an important mechanism affecting the fertilization success of males [47,48]. Females may cryptically select for males that have optimal degree of genetic similarity (or dissimilarity) with the female [8,49,50]. This indicates that polyandrous mating and sperm competition could be adaptations to avoid inbreeding. Thus, both male sperm characteristics and cryptic female choice for good (or compatible genes) may be important mechanisms predicting offspring fitness in Arctic charr (but see [51,52] for data on MHC).

We studied the benefits of polyandry by using the Arctic charr as a model species. Two main questions were: 1) What kind of benefits do females gain from polyandrous mating under sperm competition treatment? 2) Is sperm quality associated with higher offspring quality as the "good sperm" hypothesis predicts? To test these hypotheses we used a maternal half-sib breeding design, where confounding maternal investments and sire environmental effects were controlled.

## Results

### The fitness benefits of sperm competition

**Figure 1:**
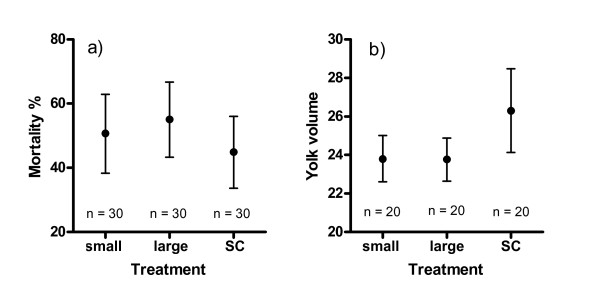
**Univariate statistic from bootstrapped samples (1000 replicates) of offspring mortality (mean % ± 95 CI) and yolk volume (mean mm^3 ^± 95 CI) across all females for different sperm treatments**. SC = sperm competition.

Offspring mortality did not differ among the three male treatments (ANOVA, *p *= 0.17, Figure 1a, Table 1), but the effect of female (total mortality percentages 15.1, 48.3 and 87.6%) and the interaction between male treatment (split plots; small, large or sperm competition) and male combination (whole plots) were statistically significant (ANOVA, *p *< 0.001 in both cases). When the single male treatments were combined and tested against the sperm competition treatment, the mortality of offspring was lower in sperm competition trials (ANOVA, F_1,9 _= 11.36, *p *= 0.01) and the effects of female as well as male combination were also significant (ANOVA, F_2,18 _= 498.75, *p *< 0.001 and F_9,11.5 _= 3.11, *p *= 0.04). Again, there was a statistically significant interaction between male treatment (sperm competition vs. single) and male combination (ANOVA, F_9,20 _= 2.92, *p *= 0.02). These results indicate offspring mortality differences between females and that the differences between small, large and sperm competition trials were not consistent across all 10 male groups (male combinations). Thus, although sperm competition group had lower mortality than in single males on average, this was not the case in all individual combinations.

**Table 1:** Partly nested split-plot ANOVA statistics for four fitness measures of the offspring.

Source	df	Mortality (%)		Body mass (g)		Total length (mm)		Yolk volume (mm^3^)	
		F	*p*	F	*p*	F	*p*	F	*p*
♀	2 (1)	414.7	**< 0.001**	329.3	**< 0.001**	2.99	0.118	128.3	**< 0.001**
mc	9 (9)	1.44	0.239	1.63	0.240	1.45	0.295	1.41	0.310
mt	2 (2)	1.94	0.173	3.31	0.058	3.28	0.059	7.17	**0.004**
♀ × mc	18 (9)	1.32	0.228	0.64	0.749	0.45	0.891	0.53	0.836
mc × mt	18 (18)	5.77	**< 0.001**	0.86	0.623	0.47	0.946	1.58	0.161

Mc = male combination (1-10), mt = male treatment (small male, large male or sperm competition, ♀ = female). Statistically significant *p*-values are indicated with boldface. Degrees of freedoms (df) for offspring body mass, total length and yolk volume are given in parentheses. Male combination comprises 10 small males, 10 large males and a sperm competition group (10 small + 10 large males simultaneously). See methods for details.

**Figure 2:**
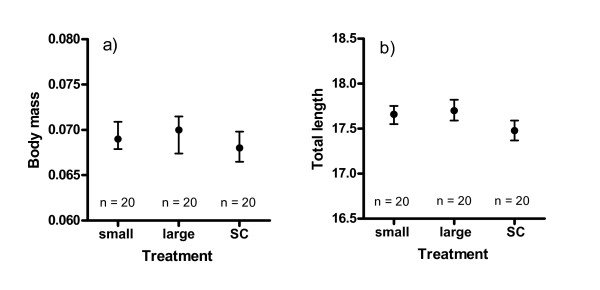
**Univariate statistic from bootstrapped samples (1000 replicates) of offspring body mass (mean g ± 95 CI) and total length (mean mm ± 95 CI) across all females for different sperm treatments**. SC = sperm competition.

The mean body mass and total length of the offspring did not differ between male treatments (ANOVA, *p *= 0.06 for both measures, Figure 2, Table 1). Female effect was significant for body mass (ANOVA, *p *< 0.001), but not for total length. The yolk sac volume varied significantly across male treatments (ANOVA, *p *= 0.004, Figure 1b). Tukey post hoc tests revealed that the size of the yolk was higher in sperm competition trials than in either of the single male trials. However, small and large males did not differ from each other in terms of the offspring yolk volume (Tukey's test). When comparisons were made between the combined single male and sperm competition groups, we found a statistically significant difference in the mean size of the offspring (ANOVA, F_1,9 _= 10.10 and 14.51, *p *= 0.01 and p = 0.004 for body mass and total length, respectively), even though the mean offspring size difference between sperm competition and single male groups was negligible (0.068 vs. 0.069 g and 17.5 vs. 17.7 mm, respectively) (Figure 2). These results can mainly be explained by the very low variance in within-group offspring body size.

### **Male and female effects on offspring fitness**

Female identity had a significant effect on all of the fitness traits we measured (ANOVA, F_2,38 _= 269.78, *p *< 0.001 for mortality, F_1,19 _= 196.07, *p *< 0.001 for body mass, F_1,19 _= 7.35, *p *= 0.014 for total length and F_1,19 _= 129.02, *p *< 0.001 for yolk reserves). Male effects were significant for offspring mortality (ANOVA, F_19,38 _= 6.52, *p *< 0.001), but not for the other three response variables (ANOVA, p > 0.15, in all cases). Small and large males did not differ from each other in any of the four offspring fitness traits (ANOVA, p > 0.40, in all cases).

### Sperm quality and offspring fitness

**Figure 3:**
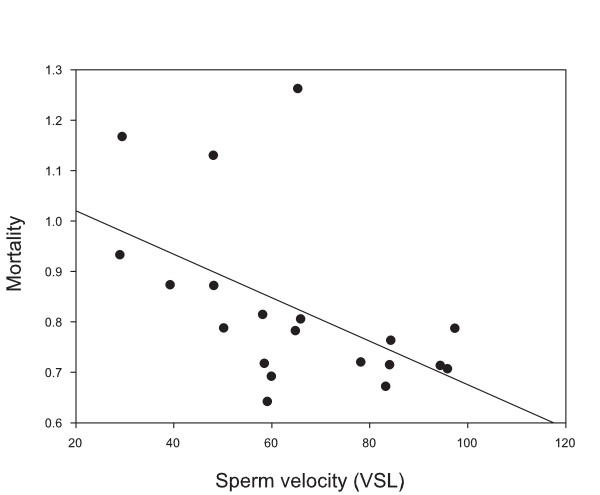
**The relationship between sperm velocity of males (VSL, *μ*m s^-1^) and offspring mortality**. The regression line is given by the equation: y = - 0.004x + 1.107.

Sperm velocity was higher in small males than in large males (ANOVA F_1,54 _= 26.53, *p *= 0.04) and there was also a female effect on sperm velocity (F_2,54 _= 28.69, *p *= 0.03). No male group (small and large males) × female interaction was found for sperm velocity (F_2,54 _= 0.08, *p *= 0.92), indicating that the effect of the female was similar for the different male groups. A statistically significant negative correlation was found between offspring mortality and sperm velocity (Pearson's correlation for VSL: whole data with three females; r = - 0.418, *p *= 0.001, n = 60, mean values of females; *r *= - 0.513, *p *= 0.021, n = 20) (Figure 3, Table 2). Sperm velocity was not associated with offspring body mass, total length or yolk volume (Table 2).

**Table 2:** Pearson correlation coefficients for the relationship between sperm velocity and offspring fitness traits.

Trait	VSL		VAP		VCL	
	*r*	*p*	*r*	*p*	*r*	*p*
mortality	- 0.418	**0.001**	- 0.422	**0.001**	- 0.416	**0.001**
body mass	- 0.244	0.128	- 0.268	0.095	- 0.292	0.067
total length	- 0.096	0.555	- 0.120	0.461	- 0.122	0.454
yolk volume	- 0.099	0.544	- 0.130	0.422	- 0.153	0.344

Statistically significant *p*-values are indicated with boldface. VSL: straight line velocity, VAP: average path velocity: VCL: curvilinear velocity.

## Discussion

In the present study, eggs from the polyandrous matings had lower mortality rates and also larger yolk reserves than the eggs from the monandrous fertilizations. It has been demonstrated that yolk mass is positively related to starvation time and thus to early performance of offspring [53,54]. Thus, our results suggest that sperm competition increases offspring fitness, not only via mortality, but that offspring may also have better ability to resist unfavourable feeding conditions during post-hatching period which are not uncommon in Arctic conditions. Although our results clearly show that polyandry is beneficial on average, they also indicate that this is not necessarily the case in all female-male combinations (see e.g. [15]).

The "good sperm" hypothesis of polyandry is believed to require the presence of additive genetic variance for male effects on offspring fitness [23]. Indeed, we found a significant male effect on offspring mortality, which indicates that survival benefits of polyandry might be explained by superior genetic quality (superior sperm) of certain male genotypes. However, male identity did not affect offspring yolk volume, although the results suggested that yolk volume was higher in offspring from polyandrous matings than offspring of either of the two males when mated monandrously. This suggests that genetic differences between males may not be sufficient to explain yolk volume differences.

Although not supported by the present results, some other studies have shown that males can indirectly contribute to offspring or yolk size [36,37,39,55]. In addition, the metabolic rate of Arctic charr eggs can vary significantly between families and this metabolic variation can have a sire component [38]. Furthermore, elevated testosterone concentration inside the eggs may increase the yolk absorption efficiency (i.e. decreases the consumption rate of the yolk), but does not affect the hatching size of the offspring [39]. If the total steroid concentration of the seminal fluid is higher when two or more males are releasing sperm simultaneously (sperm competition), it may lead to increased steroid concentrations inside the eggs. This may increase the yolk utilization efficiency [39], enabling higher growth rate with the same amount of yolk and thus bigger post-hatching yolk volume. Alternatively, if the nutrients transmitted through seminal fluid to the eggs reduce the yolk utilization rate during the incubating period, a higher proportion of the yolk may be reserved for post-hatching development.

We used the same volume of spermatozoa both in single-male and sperm-competition trials, which suggest that material benefits cannot explain the differences in yolk size. Furthermore, the maternal half-sib design allowed us to control for differences in maternal investments [56-58], which therefore did not contribute to the variation in offspring fitness observed in our study. We also minimized the biasing effect of offspring age differences on yolk reserves by sampling all the fish at the same time at the beginning of the yolk sac stage, when all the offspring had hatched. In addition, hatching was rather synchronous owing to the warm weather with most of the fish hatching within one week. These facts together with the observations that early hatching individuals are often less developed than larvae that hatch later [59,60] suggests that the observed yolk size differences in the present study were not related to the age of the offspring. As the higher yolk volume of offspring in sperm competition trials was not associated with the larger size of these fish, we cannot completely rule out the possibility that some minor differences in developmental rate between male treatments may have occurred. However, available data does not allow us to draw firm conclusions on this question at this stage, and thus, this question is an interesting avenue for further research.

As the effects of potential material benefits and maternal effects are precluded in the present study, what might explain the observed larger yolk volumes in sperm competition treatments? Theoretically, the presence of foreign sperm during sperm competition should lead to stronger competition between sperm cells and also to intensified selection for good sperm, with only the highest quality sperm (irrespective of the male) fertilizing the eggs [61]. Therefore, it seems plausible that sperm competition may intensify the selection on high quality (fast) sperm also within ejaculates (within male). In theory, this could lead to higher fitness of offspring in sperm competition compared to single matings. It is well known that only a minor proportion of sperm is capable of fertilizing the eggs [62,63]. Thus, sperm competition by males may benefit the female via two different mechanisms: first, by cryptic selection for high quality males and second, by selection for highest quality sperm within each ejaculate. However, as we did not found any association between sperm quality (velocity) and offspring yolk volume, there is also a possibility that some other, currently unknown effect may have contributed to our finding.

When the low number of founders of the study population is taken into account it seems possible that the observed fitness benefits could also be partly related to genetic incompatibility avoidance of the females [64]. Thus, polyandrous females could reduce the risk of inbreeding via cryptic selection of sperm, which would result in paternity bias towards unrelated or genetically compatible males [65,66]. As the genetic variability is extremely low in many natural Arctic charr populations [67] inbreeding avoidance can be an important mechanism explaining the evolution of polyandry in the Arctic charr. As it has been demonstrated that good genes and compatible genes may both be involved in female choice at the same time [68-70], these two mechanisms are not mutually exclusive mechanisms of sexual selection.

The "good sperm" hypothesis predicts that there should be a genetic correlation between male fertilization success and offspring viability [21,71,72]. In support of this view we found a negative association between sperm velocity and offspring mortality rate. In addition, small males had faster sperm than large males (but see [73]), probably indicating status-dependent shifts in reproductive tactics [42,74-76] (see also [77]). Sperm velocity is a good predictor of male fertilization success in the absence of sperm competition [78-81]. Additionally, in Arctic charr, sperm velocity is the most important parameter explaining variation in male fertilization success within *in vitro *sperm competition trials [44]. The positive association between sperm velocity and male sperm competition success is demonstrated also in a variety of other taxa, including the domestic fowl, *Gallus domesticus *[82], Atlantic salmon, *Salmo salar *[43] and mallards [83]. Gage et al. [43] have also shown that fertilization success of male salmon is not related to sperm number or total length and that the sperm longevity is negatively correlated to competition success (but see [84-86]). Therefore, although the relative paternity success of competing males is not known, it is likely that sperm velocity has important effects on offspring survival also under sperm competition.

## Conclusions

Different fitness benefits of polyandry documented in the present study were probably caused by different mechanisms. We demonstrate that the mortality of the embryos probably had a genetic male component and that according to "good sperm" hypothesis, sperm velocity was positively associated with offspring survival. On the other hand our results suggest that the paternal effects on offspring fitness are not necessarily under direct genetic control. Instead, in addition to potential minor developmental rate differences of offspring in different male treatments, yolk size differences may result from intensified selection pressure on high quality sperm within ejaculates or some other non-genetic factor.

## Methods

### Experimental fish

The experiment was carried out at the Taivalkoski Game and Fisheries Research station of the Finnish Game and Fisheries Research Institute. The charr descended from the Lake Inarinjärvi (northern Finland, 69°70 N, 27°29 E) population and represented a fourth hatchery generation. The number of founder families of the population started in 1982 was only 12, which indicates that inbreeding is common. In October 2007, 10 small (mean total length 50.5 cm, range 46.6-53.6 cm) and 10 large (mean total length 60.1 cm, range 55.7-70.2 cm) males as well as three females (50.8, 55.3 and 57.9 cm) were randomly selected from a common brood stock for the experiment. The males were stripped of all available milt and placed in discrete male-group-specific tanks for 12 days to prohibit spawning activity during the replenishment of their sperm reserves. This procedure ensured that all the males produced milt of similar age prior to the experiment.

### Artificial insemination

The fish were anaesthetized with buffered tricaine methanesulfonate (MS-222, Sigma^®^, Sigma Chemical Co., St. Louis, Missouri, U.S.A.) after which their total lengths and body masses were measured. After the measurements, the eggs of each female were stripped as gently as possible and divided in 30 equally large batches (approx. 100 eggs/batch) of which 10 were fertilized with the sperm of small males, 10 with the sperm of large males and 10 with the sperm of both small and large males simultaneously (sperm competition treatment). To allow polyandrous fertilization, the sperm of the both males was mixed together prior to insemination. Before milt collection, the abdomen of males was carefully wiped to prevent water contamination and activation of the sperm cells. Prior to fertilizations the spermatocrit values of each male were determined (within one hour after the milt collection) according to [42]. Same volume (i.e. approximately equal number) of spermatozoa was used in every case. In consequence, the total volume of spermatozoa/male in sperm competition trials was only half of the volume used in single male trials. The fertilization was done in Petri dishes, by injecting the sperm with micropipettes directly on the eggs. Immediately after this, 50 ml of water was poured on the Petri dish and each dish was shaken for 5 s to allow the eggs to be fertilized. The same procedure was used also in sperm competition treatments, with the sperm of the two males being injected on the eggs simultaneously. After staying 60 s in Petri dishes, fertilized eggs were randomly divided in two replicates so that the total number of egg batches was 180 (3 females × 30 batches × 2 replicates). All egg batches were incubated in independent incubating containers until hatching in the next April (approximately 180 days). Immediately after the introduction in the containers, all the eggs were photographed to determine their initial numbers.

### Sperm velocity measurements

To estimate variation in sperm velocity among study groups, we used computer-assisted sperm analysis (see [42] for details). Ovarian fluid for the analysis was obtained from the egg batches of the three females (see above) with a pipette and stored separately under the same conditions as the milt samples. Briefly, sperm activity was initially video-recorded for 40 seconds after activation, that is, from the precise moment the subsample of pure milt was exposed to 4,5 μl of ovarian fluid (2:1, OF:water) of the three females on a cooled (ca. 5°C) microscope slide (Leja Products BV, Nieuw-Vennep, The Netherlands). Recordings were made using a CCD B/W video camera (Sony XC-ST50CE PAL, Tokyo, Japan) attached to a negative phase-contrast microscope (Olympus CH30, Tokyo, Japan) with a 10× magnification objective. Video recordings were later analysed using the HTM-CEROS sperm tracker software (CEROS v.12, Hamilton Thorne Research, Beverly, MA, USA). The variables measured included: average path velocity (VAP), straight line velocity (VSL) and curvilinear velocity (VCL) [87]. The velocity estimates were based on the mean velocity of all motile cells (i.e., those exceeding the pre-determined threshold values VAP > 10 μm s^-1 ^and a VSL > 20 μm s^-1^) recorded at 10, 20, 30 and 40 s following activation. For statistical analyses, the average value of replicated measures within each male was used. As the three velocity parameters were highly correlated (Pearson, *r *> 0.95 in all cases), only VSL was used in statistical analyses.

### Mortality and size of the offspring

The number of dead eggs was counted and removed weekly, from one day after fertilization until all the fish were hatched (April 2008). Embryonic mortality was defined as a percentage of the offspring remaining from the initial number of eggs. After hatching a haphazard sample of 20 (10 × 2) offspring from each of the single male batches and 40 (20 × 2) offspring from the sperm competition batches were collected. Due to high mortality (87.3%) among one female's eggs, offspring of only two females were sampled for further (body mass, total length and yolk volume) analyses. The offspring of this female must be excluded as there was not enough fish left that reliable estimate of these offspring fitness traits could be determined (in nearly 50% of fertilization combinations the number of surviving offspring was less than five). Offspring of the other females were killed with an overdose of tricaine methanesulfonate (MS-222), photographed and their body mass was individually measured to 0.001 g precision. Prior to weighing, each fish was carefully dried using thin paper towels to prevent the possibility that extra moisture would affect the reliability of our measurements. The total length of the fish to the nearest 0.01 mm and the size of the yolk were measured from the digital images using Image-Pro Plus 3.0 graphic software (Media Cybernetics, Inc., Silver Spring, MD, USA). The volume of the yolk was calculated according the equation for a prolate spheroid: V = 0.5236 × length × height^2 ^(e.g. [88]). All the fish were preserved in 95% ethanol for later paternity analyses (results are presented elsewhere). The study was carried out according to Finnish legislation.

### Statistical analyses

We used a partly nested split-plot ANOVA to analyze the differences in mortality and the offspring size variables (body mass, total length and yolk volume) between single male and sperm competition trials. Females were used as blocks in the analysis. Each block was divided into 10 whole plots (10 small, 10 large and 10 small + large males), where each plot include one small male, one large male and one sperm competition treatment. Each whole plot was divided into three split plots; large male, small male and sperm competition (i.e. three male treatments were nested within 10 male combinations). Because of high mortality in some male-female combinations missing values were also present. Therefore, we used the mean values of the two replicates to achieve balanced data in all analyses (i.e. when missing values were present mean values were replaced with the value of the other replicate). To study whether polyandrous females have a higher fitness than the average fitness of single mating females, we combined large and small male groups and compared the mean mortality, body mass and total length of the offspring between sperm competition versus single male trials.

The effects of individual males and females on offspring fitness (mortality, body mass, total length and yolk volume) were studied using two-way ANOVAs with three (or two) females and 20 males as random factors and the mean values of the two replicates as data points for each female-male combination. To reveal individual male effects, only single-male trials were used in these analyses. Furthermore, the differences between small and large male with respect to offspring mortality, body mass, total length, yolk volume and sperm VSL were tested using two-way ANOVA, with male group (small vs. large) as a fixed factor and female as a random factor.

The relationships between sperm quality and offspring fitness were studied with Pearson's correlation analyses. Prior to statistical analyses, the mortality percentages were transformed with the arcsine-square-root transformation to achieve normality. The fulfillment of the assumptions of all statistical tests was determined according to [89].

## Authors' contributions

RK, GR, LF and NP designed the experiments. Experiments were carried out with the cooperation of all authors. JK, MJ, GR and NT measured the fitness parameters. JK and MJ performed the statistical analyses and JK wrote the paper. All authors commented on the manuscript and approved the final version.
